# Potential of a Second-Generation Dual-Layer Spectral CT for Dose Calculation in Particle Therapy Treatment Planning

**DOI:** 10.3389/fonc.2022.853495

**Published:** 2022-04-20

**Authors:** Friderike K. Longarino, Antonia Kowalewski, Thomas Tessonnier, Stewart Mein, Benjamin Ackermann, Jürgen Debus, Andrea Mairani, Wolfram Stiller

**Affiliations:** ^1^ Clinical Cooperation Unit Radiation Oncology, German Cancer Research Center (DKFZ), Heidelberg, Germany; ^2^ Department of Radiation Oncology, Heidelberg University Hospital, Heidelberg, Germany; ^3^ Department of Physics and Astronomy, Heidelberg University, Heidelberg, Germany; ^4^ Translational Radiation Oncology, German Cancer Research Center (DKFZ), Heidelberg, Germany; ^5^ Department of Physics, Simon Fraser University, Burnaby, BC, Canada; ^6^ Heidelberg Ion Beam Therapy Center (HIT), Heidelberg, Germany; ^7^ Heidelberg Institute of Radiation Oncology (HIRO), National Center for Radiation Research in Oncology (NCRO), Heidelberg, Germany; ^8^ National Center for Tumor Diseases (NCT), Heidelberg, Germany; ^9^ German Cancer Consortium (DKTK), Core Center Heidelberg, Heidelberg, Germany; ^10^ Medical Physics, National Center of Oncological Hadrontherapy (CNAO), Pavia, Italy; ^11^ Diagnostic and Interventional Radiology (DIR), Heidelberg University Hospital, Heidelberg, Germany

**Keywords:** dual-layer spectral CT, particle therapy, Spectral CT 7500, stopping power ratio, range uncertainty, treatment planning

## Abstract

In particle therapy treatment planning, dose calculation is conducted using patient-specific maps of tissue ion stopping power ratio (SPR) to predict beam ranges. Improving patient-specific SPR prediction is therefore essential for accurate dose calculation. In this study, we investigated the use of the Spectral CT 7500, a second-generation dual-layer spectral computed tomography (DLCT) system, as an alternative to conventional single-energy CT (SECT) for patient-specific SPR prediction. This dual-energy CT (DECT)-based method allows for the direct prediction of SPR from quantitative measurements of relative electron density and effective atomic number using the Bethe equation, whereas the conventional SECT-based method consists of indirect image data-based prediction through the conversion of calibrated CT numbers to SPR. The performance of the Spectral CT 7500 in particle therapy treatment planning was characterized by conducting a thorough analysis of its SPR prediction accuracy for both tissue-equivalent materials and common non-tissue implant materials. In both instances, DLCT was found to reduce uncertainty in SPR predictions compared to SECT. Mean deviations of 0.7% and 1.6% from measured SPR values were found for DLCT- and SECT-based predictions, respectively, in tissue-equivalent materials. Furthermore, end-to-end analyses of DLCT-based treatment planning were performed for proton, helium, and carbon ion therapies with anthropomorphic head and pelvic phantoms. 3D gamma analysis was performed with ionization chamber array measurements as the reference. DLCT-predicted dose distributions revealed higher passing rates compared to SECT-predicted dose distributions. In the DLCT-based treatment plans, measured distal-edge evaluation layers were within 1 mm of their predicted positions, demonstrating the accuracy of DLCT-based particle range prediction. This study demonstrated that the use of the Spectral CT 7500 in particle therapy treatment planning may lead to better agreement between planned and delivered dose compared to current clinical SECT systems.

## 1 Introduction

The central goal of modern radiotherapy is the delivery of maximum radiation dose to tumors while minimizing radiation dose to healthy surrounding tissue. Particle therapy offers promising advancements in this regard (1), thanks to the favourable depth-dose curve of charged particles compared to conventional photon beams (X-rays) (2). However, to take full advantage of the benefits of particle therapy, it is essential to have precise, accurate, and patient-specific predictions of particle ranges within the body (3). For clinical treatment planning, predicted particle ranges are calculated from ion stopping power ratio (SPR) maps, which are in turn derived from patient computed tomography (CT) data. At present, CT numbers (CTNs) from single-energy CT (SECT) images are converted to SPR values using a generic, empirically validated conversion function called a Hounsfield look-up table (HLUT) (**Supplementary Figure 1**). This approach to SPR prediction is a main source of beam range uncertainty, as HLUTs do not account for degeneracies between CTN and SPR values, nor for variability in tissue composition between patients (4–8).

Recently, dual-energy CT (DECT), clinically introduced for diagnostic imaging in 2006 (9), has been investigated as an alternative to SECT. In DECT, two CT data sets are acquired using different X-ray spectra, enabling the generation of relative electron density (ED) and effective atomic number (EAN) maps (10). From ED and EAN data, SPR values can be calculated through the Bethe equation without the need for a pre-defined HLUT (10, 11). Both theoretical (5, 10) and experimental (5, 12–22) studies have shown DECT to improve SPR prediction accuracy over SECT. Several imaging techniques and modalities exist to achieve DECT results, including dual-spiral, dual-source, rapid kV switching, twin-beam, and dual-layer technologies (7) (**Supplementary Tables 1**, **2**). Of these, dual-layer spectral CT (DLCT) employs a double-layer detector to simultaneously acquire high- and low-energy X-ray data (23). This avoids exposing the patient to additional radiation (21), and achieves synchronicity between the low- and high-energy data acquisitions over the full scan field-of-view, facilitating the imaging of moving organs (24).

At present, the SPR prediction accuracy of DLCT has only been investigated using the Philips IQon Spectral CT (Philips Healthcare, Best, The Netherlands) (21, 22, 24–27). Here, we investigate the SPR prediction accuracy of the new Philips Spectral CT 7500 (Philips Healthcare, Best, The Netherlands), commissioned at the Heidelberg University Hospital (Germany) for diagnostic use in February 2021 and officially released in May 2021. This scanner offers advantages over the Philips IQon Spectral CT, including a new high-performance patient table, a larger (anatomical) detector coverage enabling a greater number of simultaneously acquired slices per rotation (up to 256 versus 128), and a larger bore size (**Supplementary Table 3**). The large bore size of 800 mm allows for easier access to patients, and better accommodation of patient accessories and obese patients. Furthermore, the Philips Spectral CT 7500 allows the generation of spectral results at 100, 120, and 140 kV_p_.

We seek to validate the Philips Spectral CT 7500 for particle therapy treatment planning by conducting a thorough analysis of its SPR prediction accuracy for both tissue-equivalent materials and common non-tissue implants. To our knowledge, this is the first study conducted on second-generation DLCT systems (i.e., Spectral CT 7500) and here we focus specifically on applications to particle therapy. We employed the same methodology as in the relevant publications on the first-generation system (22, 25, 27) in order to allow for direct comparability to results from prior studies. Furthermore, we perform end-to-end analyses for proton, helium ion, and carbon ion therapies with anthropomorphic head and pelvic phantoms.

## 2 Materials and Methods

### 2.1 CT Image Acquisition and Reconstruction

All images were acquired using the Philips Spectral CT 7500 scanner (Philips Healthcare, Best, The Netherlands) at the Heidelberg University Hospital with a standardized head or body protocol at 120 kV_p_. The image acquisition settings and reconstruction parameters for head and body protocols are specified in **Supplementary Table 4**, and are based on current state-of-the-art clinical protocols used for particle therapy planning at the Heidelberg Ion Beam Therapy Center (HIT, Germany). Both SECT and DLCT image data are automatically generated from the same raw data set for each acquisition on the Spectral CT 7500 scanner, enabling a direct comparison of the two techniques.

Image reconstruction was performed using the iDose^4^ algorithm at levels 0, 3, and 6 (Philips Healthcare, Best, The Netherlands). The iDose^4^ algorithm uses a hybrid iterative reconstruction technique to reduce image noise, and has levels ranging from 0 to 6, where higher levels correspond to greater noise reduction. In this context, an iDose^4^ level of 0 corresponds to conventional filtered back-projection image reconstruction. For imaging of metallic materials, the Philips orthopedic metal artifact reduction algorithm (O-MAR) (Philips Healthcare, Best, The Netherlands) was also applied.

### 2.2 SPR Prediction and Validation in Geometric Phantoms

**Figure 1:**
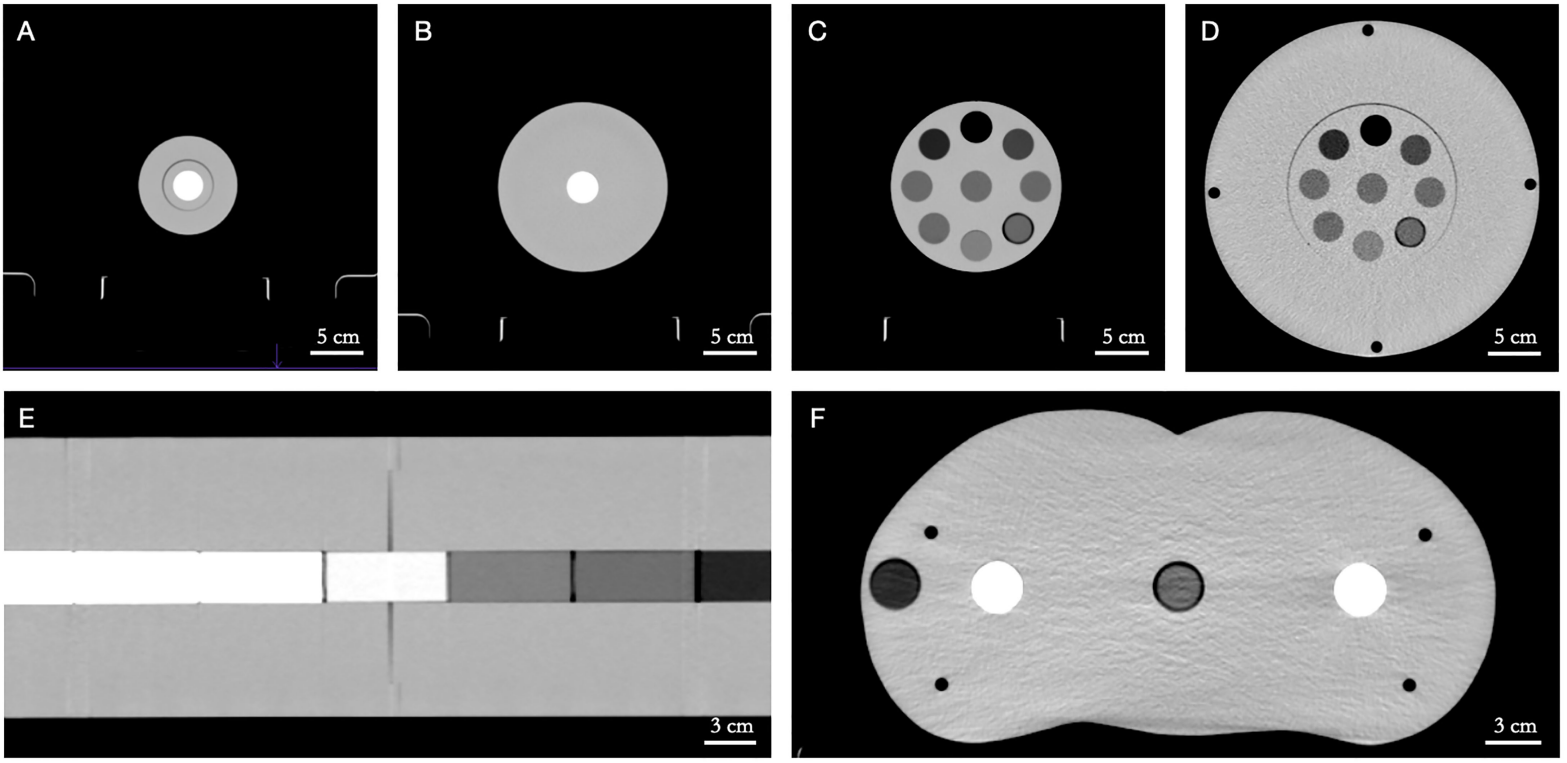
Custom polymethyl methacrylate (PMMA) phantoms with tissue-equivalent inserts in axial view. **(A)** LCT (“long cylinder thin”) phantom, **(B)** LC (“long cylinder”) phantom, **(C)** SC (“short cylinder”) phantom, **(D)** SCB (“short cylinder big”) phantom, **(E)** LC (“long cylinder”) phantom (in coronal view), **(F)** pelvis phantom. Window level/window width = 40/400 HU.

The SPR prediction accuracy of the Philips Spectral CT 7500 scanner was first investigated using a number of custom cylindrical polymethyl methacrylate (PMMA) phantoms with tissue-equivalent inserts spanning the range of clinically relevant CTNs (**Figure 1**). Five PMMA phantoms were used to simulate different patient sizes: two one-bore cylinders of height 46.0 cm and radius 5.0 cm (“LCT”, “long cylinder thin”) and 8.0 cm (“LC”, “long cylinder”), two nine-bore cylinders of height 10.0 cm and radius 8.0 cm (“SC”, “short cylinder”) and 16.0 cm (“SCB”, “short cylinder big”), and a roughly human-shaped pelvis. Thirteen tissue-equivalent cylindrical inserts (Gammex Electron Density CT Phantom 467, Gammex-RMI, Middleton, WI, USA) of height 7.0 cm and radius 1.4 cm were used as bore inserts: cortical bone, CB2 50%, CB2 30%, inner bone, muscle, brain, adipose, true water, liver, solid water, breast, bone mineral, and lung. Reference SPR values of these inserts were determined experimentally at HIT by measuring the range shift of a carbon ion beam in a water absorber (Peakfinder Water Column, PTW-Freiburg, Freiburg, Germany). Carbon ions were used for the measurement due to their sharper Bragg peak, reduced lateral scattering, and reduced range straggling compared to protons (22). The inserts were placed in the phantoms in specific configurations to minimize artifacts caused by the high-density bone-equivalent inserts (14).

Furthermore, a selection of materials commonly found in non-tissue implants were scanned for SPR prediction. The metals aluminum and titanium and a carbon/PEEK-titanium composite (icotec ag, Altstätten, Switzerland) were imaged in the LC phantom, along with the special materials PMMA, TECAFORM^®^ and TECAPEEK^®^ (Ensinger GmbH, Nufringen, Germany), and Teflon™ (The Chemours Company, Wilmington, DE, USA). In addition, PALACOS^®^ R + G bone cement (Heraeus, Hanau, Germany) was imaged in a water bath.

#### 2.2.1 Calculation of Predicted SPR Values Based on Quantitative DLCT Data

Predicted SPR values were calculated from DLCT-generated ED and EAN maps using the Bethe equation neglecting higher order correction terms (11), as described in Faller et al. (22). The mean excitation energy (I-value) of the tissue was calculated from EAN data using the method outlined in Yang et al. (10). The I-value of water was set to 78.73 eV, consistent with the values proposed by Bär et al. (28) and the International Commission on Radiation Units and Measurements (29). A fixed particle kinetic energy of 100 MeV per nucleon was assumed, as recommended by Inaniwa & Kanematsu (30), since the energy dependence of SPR prediction is minimal in the therapeutic range (31).

#### 2.2.2 Calculation of Predicted SPR Values Based on Conventional SECT Image Data

For each of the two imaging protocols (head and body), an HLUT was generated from 120 kV_p_ SECT image data acquired using the given protocol. A two-parameter stoichiometric parametrization (11, 32) was applied to generate the HLUT, following the current clinical protocol at HIT (33). The generated HLUT was then used to convert CTNs to SPR values (**Supplementary Figure 1**).

#### 2.2.3 Assessment of DLCT- and SECT-Based SPR Predictions

Predicted SPR values of cylindrical phantom inserts were extracted for analysis using circular regions-of-interest (ROIs) with a size of ~70% of the inserts’ cross-sectional diameters. This strategy avoided possible artifacts caused by gradient effects to the surrounding PMMA near the insert–phantom boundary. ROI slices towards both ends of the inserts were also excluded for similar reasons. Predicted SPR values of the PALACOS^®^ R + G bone cement, imaged in a water bath, were extracted for analysis using a similar method, where ROIs were evaluated at cross-sectional locations along the longest axis of the bone cement sample.

The agreement of predicted SPR values (SPR_pre_) with reference values (SPR_ref_) was quantified using relative residuals, defined as


$$relativeresidual=\frac{SPR_{pre}-SPR_{ref}}{SPR_{ref}}\cdot 100\%$$

For each phantom–protocol combination, the mean overall relative residual was computed using the formula


$$meanoverallrelativeresidual=\frac{1}{N}\sum_{i=1}^{N}\left|relativeresidual\right|$$

Additionally, for each phantom–protocol combination, the root-mean-square error (RMSE) and Pearson’s correlation coefficient (*r*) between predicted and reference SPR values were determined, using the formulas


$$RMSE=\sqrt{\frac{1}{N}\sum_{i=1}^{N}{\left(SPR_{pre,i}-SPR_{ref,i}\right)}^{2}}$$

and


$$r=\frac{\Sigma_{i=1}^{N}(SPR_{ref,i}-\bar{SPR_{ref}})(SPR_{pre,i}-\bar{SPR_{pre}})}{\sqrt{\Sigma_{i=1}^{N}{\left(SPR_{ref,i}-\bar{SPR_{ref}}\right)}^{2}}\sqrt{\Sigma_{i=1}^{N}{\left(SPR_{pre,i}-\bar{SPR_{pre}}\right)}^{2}}}$$

respectively. In both cases, N is the number of cylindrical inserts in a given phantom. In the Pearson’s correlation coefficient formula, the bars represent arithmetic means.

Finally, predicted SPR values were fitted to reference values using linear regression, with parameters *α* and *δ*:


$$SPR_{pre}=\alpha \cdot SPR_{ref}+\delta$$

Pearson’s correlation coefficient (*r*) and linear regression fitting parameters (*α* and *δ*) were used to quantify the agreement of DLCT- and SECT-based SPR predictions with measured reference values.

#### 2.2.4 Evaluation of DLCT-Based Mass Density Calculation

We implemented and evaluated the DEEDZ-MD method proposed by Saito (34) to derive mass density (ρ) from DLCT data. ρ was calculated from DLCT-based ED (ρ_e_) and EAN (Z_eff_) values, with the EAN of water being Z_eff,w_:


$$\rho =\rho_{e}+\rho_{e}\sum_{n=0}^{2}e_{n}{\left\{{\left(\frac{Z_{eff}}{Z_{eff,w}}\right)}^{m}-1\right\}}^{n}$$

The value of m was set to 3.3, as determined in Saito & Sagara (35), and the same human tissue-specific parameters (e_n_) as obtained in Saito (34) were employed.

### 2.3 Treatment Planning and Dosimetric Validation With Anthropomorphic Head and Pelvic Phantoms

The clinical benefits of SPR prediction based on DLCT data were investigated and compared with the currently applied SECT approach by using tissue-equivalent anthropomorphic head (Proton Therapy Dosimetry Head, Model 731-HN) and pelvic (Virtual Human Male Pelvis Phantom, Model 801-P) phantoms (Computerized Imaging Reference Systems, Inc. (CIRS), Norfolk, VA, USA).

**Figure 2:**
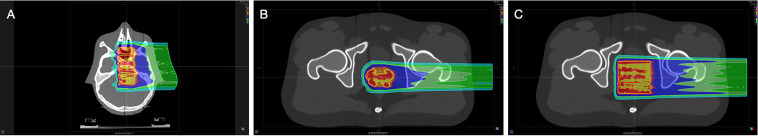
Proton therapy treatment plans designed with the RayStation Treatment Planning System. **(A)** Head phantom with an 8 x 8 x 3 cm^3^ target volume, **(B)** pelvic phantom with a prostate-like target volume of 52 cm^3^, **(C)** pelvic phantom with a 6 x 6 x 6 cm^3^ target volume.

Treatment planning optimizations with a dose grid of 1 mm were performed with RayStation Treatment Planning System v10 (RaySearch Laboratories AB, Stockholm, Sweden), using the Monte-Carlo dose engine for proton beams and the pencil beam dose engine for helium and carbon ion beams (**Figure 2**). The target position for each anthropomorphic phantom was selected such that it was located underneath multiple different tissue-equivalent layers, in order to test the various range prediction methods in heterogenous conditions. For the head phantom, an 8 x 8 x 3 cm^3^ target volume located at the mid-head was optimized for a physical dose of 1 Gy (**Figure 2A**). For the pelvic phantom, two target types were optimized for a physical dose of 1 Gy: a prostate-like geometry of 52 cm^3^ (**Figure 2B**) and a 6 x 6 x 6 cm^3^ target volume (**Figure 2C**).

Treatment planning was initially performed with a conventional clinically-employed SECT scanner (SOMATOM Confidence, Siemens Healthcare GmbH, Erlangen, Germany) with a CT resolution of 0.977 x 0.977 x 1 mm^3^ (head)/0.977 x 0.977 x 2 mm^3^ (pelvis). Following plan optimization, forward dose calculations were performed on two additional (image) datasets from the Philips Spectral CT 7500: one using the SECT approach and one using the DLCT approach for SPR prediction.

Dosimetric measurements were acquired at HIT with the OCTAVIUS^®^ 1000SRS P (PTW, Freiburg, Germany) prototype 2D ionization chamber array detector for proton, helium ion, and carbon ion beam treatment plans, as described in previous works (36). For both phantoms, measurements were performed in the high-dose area and at different positions along the distal edge. For the head phantom, irradiation was performed using the gantry at an angle of 0° with the half-head phantom placed on top of the OCTAVIUS^®^ detector (**Supplementary Figure 2A**). For the pelvic phantom, irradiation was performed using the horizontal beam line with the half-pelvic phantom placed in front of the OCTAVIUS^®^ detector (**Supplementary Figure 2B**).

Dose distributions were compared using a 3D gamma analysis (37) for local calculation with a passing criterion of 3%/1.5 mm using a low dose cut-off of 5% of the maximum dose.

## 3 Results

### 3.1 CT Image Acquisition and Reconstruction

CT (image) data acquired using the head and body protocols produced similarly accurate SPR predictions (**Supplementary Tables 5**, **6**). As such, all reports of SPR prediction accuracy for the remainder of the study are based on CT images acquired using the body protocol, unless otherwise specified.

Similarly, the iDose^4^ level used in image reconstruction was found to have no significant effect on the accuracy and standard deviation of predicted SPR values (**Supplementary Tables 5**, **6**). Therefore, all results reported for the remainder of the study are based on CT (image) data reconstructed using iDose^4^ level 0 (that is, with minimum additional iterative post-processing), unless otherwise specified.

### 3.2 SPR Prediction and Assessment in Geometric Phantoms

**Figure 3:**
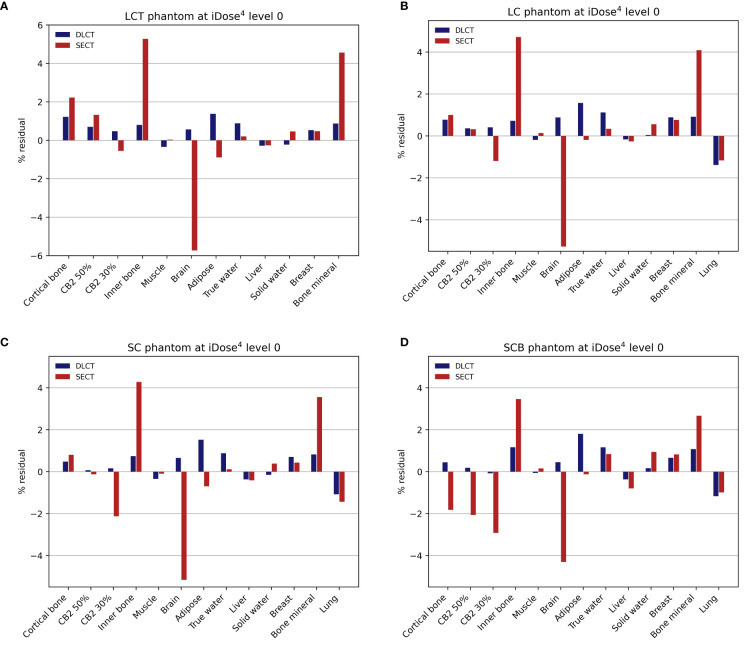
Relative residuals for DLCT- and SECT-based SPR predictions compared to reference values. **(A)** LCT (“long cylinder thin”) phantom, **(B)** LC (“long cylinder”) phantom, **(C)** SC (“short cylinder”) phantom, and **(D)** SCB (“short cylinder big”) phantom. The measurement of the LCT phantom was performed without the lung insert, as the lung insert did not fit into the LCT phantom due to its slightly larger diameter. Note the different scaling of the y-axis in **(A)**.

For tissue surrogates, SPR values predicted using DLCT were consistently closer to reference values than SPR values predicted using SECT in all five phantoms (**Figure 3**; **Tables 1**, **2**). Pearson’s correlation coefficient (*r*) and linear regression fitting parameters (*α* and *δ*) confirmed higher agreement between measured and DLCT-predicted SPR values compared to SECT-predicted SPR values (**Tables 1**, **2**). For consistency over all tissue-equivalent inserts, we focus solely on the LCT, LC, SC, and SCB phantoms for the remainder of the study, as not all inserts were imaged in the pelvis phantom.

**Table 1:** Accuracy of DLCT-based SPR predictions across five different PMMA phantoms: LCT (“long cylinder thin”) phantom, LC (“long cylinder”) phantom, SC (“short cylinder”) phantom, SCB (“short cylinder big”) phantom, and a roughly human-shaped pelvis.

Phantom	LCT	LC	SC	SCB	Pelvis
Mean overall relative residual	0.688	0.725	0.613	0.675	0.816
RMSE	0.0093	0.0084	0.0056	0.0067	0.0116
*r*	0.9996	0.9998	0.9998	0.9997	0.9998
*α*	1.018	1.011	1.005	1.004	0.984
*δ*	-0.014	-0.006	-0.002	0.000	0.010

The table shows the mean overall relative residual, root-mean-square error (RMSE), Pearson's correlation coefficient (r), and linear regression fitting parameters (α and δ).

**Table 2:** Accuracy of SECT-based SPR predictions across five different PMMA phantoms: LCT (“long cylinder thin”) phantom, LC (“long cylinder”) phantom, SC (“short cylinder”) phantom, SCB (“short cylinder big”) phantom, and a roughly human-shaped pelvis.

Phantom	LCT	LC	SC	SCB	Pelvis
Mean overall relative residual	1.833	1.538	1.514	1.685	0.859
RMSE	0.0307	0.0255	0.0243	0.0245	0.0101
*r*	0.9908	0.9956	0.9958	0.9962	0.9996
*α*	1.046	1.012	1.007	0.965	1.003
*δ*	-0.044	-0.009	-0.008	0.033	-0.005

The table shows the mean overall relative residual, root-mean-square error (RMSE), Pearson's correlation coefficient (r), and linear regression fitting parameters (α and δ).

For the LCT phantom, DLCT-based SPR prediction had a mean overall relative residual of 0.7% (range: [-0.3, 1.4]%) while SECT-based SPR prediction had a mean overall relative residual of 1.8% (range: [-5.7, 5.3]%) (**Figure 3A**). For the LC phantom, DLCT-based SPR prediction had a mean overall relative residual of 0.7% (range: [-1.4, 1.6]%) while SECT-based SPR prediction had a mean overall relative residual of 1.5% (range: [-5.3, 4.7]%) (**Figure 3B**). For the SC phantom, DLCT-based SPR prediction had a mean overall relative residual of 0.6% (range: [-1.1, 1.5]%) while SECT-based SPR prediction had a mean overall relative residual of 1.5% (range: [-5.2, 4.3]%) (**Figure 3C**). Finally, for the SCB phantom, DLCT-based SPR prediction had a mean overall relative residual of 0.7% (range: [-1.2, 1.8]%) while SECT-based SPR prediction had a mean overall relative residual of 1.7% (range: [-4.3, 3.5]%) (**Figure 3D**). Across all four phantoms, the average mean overall relative residual was 0.7% for DLCT-based SPR prediction and 1.6% for SECT-based SPR prediction.

Accuracies of DLCT- and SECT-based SPR predictions across different non-tissue implant materials are listed in **Table 3**. DLCT substantially outperformed SECT in predicting SPR values for all non-tissue materials. For the metals aluminium and titanium, SPR prediction accuracy was similar with and without the metal artifact reduction algorithm O-MAR. The HLUT derived in the SECT-based approach is shown together with the eight non-tissue implant materials in **Supplementary Figure 1**.

**Table 3:** Accuracy of SECT- and DLCT-based SPR predictions across different non-tissue materials.

Material	Relative residual SECT	Relative residual DLCT
Aluminium	-7.5	6.9
Carbon/PEEK-titanium composite	-16.8	1.0
Palacos bone cement	45.0	8.1
PMMA	-6.7	1.3
Tecaform	-13.7	2.2
Tecapeek	-11.0	1.7
Teflon	-19.8	4.4
Titanium	-28.0	18.4

Experimental validation of the DEEDZ-MD method for determining mass density was performed using the tissue-equivalent inserts in the SC phantom, yielding a relative mean deviation of -1.4% compared to the vendor’s provided mass density data (Gammex Electron Density CT Phantom 467, Gammex-RMI, Middleton, WI, USA).

For the SC phantom, the effect of lowering the tube current-time product on SPR prediction accuracy was also investigated. The tube current-time product was lowered from 300 mAs to 250 mAs and 200 mAs with no adverse effect on the SPR prediction accuracy and its standard deviation. Increasing the tube voltage from 120 kV_p_ to 140 kV_p_ while using a tube current-time product of 200 mAs resulted in approximately the same volume CT dose index (CTDI_vol_) as for the standard clinical protocol. DLCT-based SPR prediction using these CT acquisition settings (140 kV_p_/200 mAs) had a mean overall relative residual of 0.6%, which is equal to that of the 120 kV_p_/300 mAs protocol.

### 3.3 Treatment Planning and Dosimetric Validation With Anthropomorphic Head and Pelvic Phantoms

3D gamma analysis (3%/1.5 mm) using local calculation between SECT- and DLCT-based dose distributions and dosimetric measurements acquired with the OCTAVIUS^®^ ionization chamber array using the anthropomorphic head phantom revealed substantial agreement between measured and calculated dose distributions (**Tables 4**, **5**). For all three ion types, DLCT-based dose distributions showed higher 3D gamma passing rates compared to SECT-based dose distributions.

**Table 4:** 3D gamma passing rates (3%/1.5 mm) using local calculation between SECT- and DLCT-based dose distributions and dosimetric measurements acquired with the OCTAVIUS^®^ ionization chamber array using the anthropomorphic head phantom.

3D gamma passing rate in %								
^1^H			^4^He			^12^C		
Measurement position	SECT	DLCT	Measurement position	SECT	DLCT	Measurement position	SECT	DLCT
High-dose area, position A	98.6	98.8	High-dose area, position A	97.8	97.9	High-dose area, position A	96.9	97.0
High-dose area, position B	95.8	97.7	High-dose area, position B	95.7	99.4	92% dose fall-off	88.9	97.0
87% dose fall-off	92.3	99.6	72% dose fall-off	87.7	94.8	70% dose fall-off	75.7	78.3
72% dose fall-off	93.5	97.4	55% dose fall-off	81.5	84.6	53% dose fall-off	80.4	86.5

Four different depths were investigated, with two of the depths for ^1^H and ^4^He being in the high-dose area (positions A and B), while the second depth for ^12^C was already in the dose fall-off.

**Table 5:** 3D gamma passing rates (3%/1.5 mm) using local calculation between SECT- and DLCT-based dose distributions and dosimetric measurements acquired with the OCTAVIUS^®^ ionization chamber array using the anthropomorphic pelvic phantom.

3D gamma passing rate in %								
^1^H			^4^He			^12^C		
Measurement position	SECT	DLCT	Measurement position	SECT	DLCT	Measurement position	SECT	DLCT
High-dose area (prostate-like geometry)	97.2	99.3	High-dose area (prostate-like geometry)	99.4	99.4	High-dose area (prostate-like geometry)	99.3	99.5
High-dose area, position A (cubic target volume)	95.7	98.1	High-dose area, position A (cubic target volume)	99.4	99.4	High-dose area, position A (cubic target volume)	93.3	93.7
95% dose fall-off (cubic target volume)	81.8	100.0	95% dose fall-off (cubic target volume)	83.2	94.3	High-dose area, position B (cubic target volume)	92.6	98.1
83% dose fall-off (cubic target volume)	85.9	100.0	81% dose fall-off (cubic target volume)	83.0	92.0	94% dose fall-off (cubic target volume)	94.8	97.9
64% dose fall-off (cubic target volume)	86.9	99.9	63% dose fall-off (cubic target volume)	82.0	91.1	80% dose fall-off (cubic target volume)	96.7	97.6

For the cubic target volume, four different depths were investigated, with two of the depths for ^12^C being in the high-dose area (positions A and B), while the second depth for ^1^H and ^4^He was already in the dose fall-off.

For the head phantom, the 3D gamma passing rates (3%/1.5 mm) were 98.8% (^1^H), 97.9% (^4^He), and 97.0% (^12^C) using DLCT for the high-dose area of the target volume. For DLCT, the measured distal position of the 72% (^1^H)/55% (^4^He)/53% (^12^C) dose level of the target volume was within 1 mm of the predicted distal position of the respective dose level with 3D gamma passing rates (3%/1.5 mm) of 97.4% (^1^H), 84.6% (^4^He), and 86.5% (^12^C) (**Table 4**).

For the pelvic phantom, the 3D gamma passing rates (3%/1.5 mm) were 99.3% (^1^H), 99.4% (^4^He), and 99.5% (^12^C) using DLCT for the high-dose area of the prostate-like geometry and 98.1% (^1^H), 99.4% (^4^He), and 93.7% (^12^C) for the high-dose area of the cubic target volume. For DLCT, the measured distal position of the 64% (^1^H)/63% (^4^He)/80% (^12^C) dose level of the cubic target volume was within 1 mm of the predicted distal position of the respective dose level with 3D gamma passing rates (3%/1.5 mm) of 99.9% (^1^H), 91.1% (^4^He), and 97.6% (^12^C) (**Table 5**).

## 4 Discussion

### 4.1 Key Findings

In this study, we performed a thorough analysis of the use of the Philips Spectral CT 7500 DLCT system for SPR prediction in particle therapy treatment planning. For this purpose, we experimentally verified DLCT-based SPR prediction accuracy and its impact on dose calculation in particle therapy planning with a Spectral CT 7500 scanner using tissue surrogates and non-tissue implant materials as well as anthropomorphic head and pelvic phantoms. To our knowledge, this is the first study to investigate this second-generation DLCT system for application in particle therapy. Moreover, this study presents the first dosimetric validation of DECT-based dose prediction using anthropomorphic phantoms for helium and carbon ion treatment plans. It is important to investigate DECT-based SPR prediction for helium and carbon ions since the impact of range uncertainty for these ion beams may lead to sizeable biological dose deviation, given the sharp gradients of linear energy transfer (LET) and relative biological effectiveness (RBE) end-of-range (38).

For tissue-equivalent materials, DLCT exhibited greater SPR prediction power, in general, compared to SECT with mean overall relative residuals of 0.6–0.7% for DLCT-based predictions and 1.5–1.8% for SECT-based predictions (**Figure 3**; **Tables 1**, **2**). Ranges represent the variability introduced by four different phantom geometries. Furthermore, there are individual differences in the tissue-equivalent inserts, as discussed in Faller et al. (22). The larger residuals of SECT-based SPR predictions for certain tissue-equivalent inserts (i.e., bone mineral, brain, and inner bone substitutes) (**Figure 3**) may in part result from differences between the elemental composition of the tissue surrogate inserts and their real tissue counterparts (39).

In clinical practice, many complicating factors to straight-forward SPR prediction exist, such as the presence of artifact-inducing implants in patients. Therefore, we also validated the use of DLCT for SPR prediction in eight common non-tissue implant materials. DLCT again outperformed SECT, although relative residuals for both approaches were significantly greater than those for tissue-equivalent materials: 1.0–18.4% for DLCT-based predictions, and -6.7%–45.0% for SECT-based predictions (**Table 3**). To illustrate the importance of SPR prediction for non-tissue implant materials, we consider the example of PALACOS^®^ R + G bone cement. This common component of artificial joints is made mostly of PMMA and zirconium dioxide. Despite the presence of zirconium dioxide, a high-atomic-number material, the SPR of PALACOS^®^ R + G is relatively low. The resulting uncertainty in SPR prediction can lead to a particle range deviation of several millimeters when using SECT-based treatment planning. Even if DECT is not implemented for quantitative SPR prediction in clinical practice, spectral image data could still be used to better differentiate between normal tissues and non-tissue implant materials and to help identify properties relating to the stopping power of non-tissue implant materials for contouring and SPR override. For example, using known ED and EAN data sets of commonly used implant materials, comparisons can be performed to quantify relevant physical properties to predict stopping power for unknown materials.

Furthermore, we used the Philips Spectral CT 7500 to experimentally validate the DEEDZ-MD method for determining mass density proposed by Saito (34). Our results showed a mean deviation of -1.4% from the reference value, which is similar to the -1.34% deviation reported by Saito (34). Future work may be dedicated to exploring treatment planning possibilities using mass density data.

We also found a result which suggests that tube current-time product can be lowered by 100 mAs in a simple geometric PMMA phantom without adverse effects on SPR prediction accuracy. Minimizing CT acquisition dose is an important component of CT research, particularly in fields with large pediatric contingents, such as particle therapy.

Finally, we demonstrated the feasibility of using the Philips Spectral CT 7500 to improve particle range prediction by irradiating anthropomorphic head and pelvic phantoms. We showed that dose distributions of DLCT-based treatment plans showed greater agreement with ionization chamber-measured dose distributions than dose distributions of SECT-based treatment plans for proton, helium ion, and carbon ion beams (**Tables 4**, **5**).

### 4.2 Comparison to Previous Work

DLCT-based SPR prediction accuracy was previously investigated at HIT using many of the same phantoms and tissue-equivalent inserts as in this study, but with the Philips IQon Spectral CT (22) (**Supplementary Table 2**). DLCT-based SPR prediction in that study yielded mean overall relative residuals of 0.6–0.9%, compared to the 0.6–0.7% reported here. These results indicate that the SPR prediction accuracy of the Philips Spectral CT 7500 is on par with that of the Philips IQon Spectral CT. However, the Philips Spectral CT 7500 provides numerous other advantages over the Philips IQon Spectral CT (**Supplementary Table 3**).

A related study using the Philips IQon Spectral CT reported similar SPR prediction accuracy results using mono-energetic images and the same inserts for calibration and evaluation (RMSE=0.6%) (25). Moreover, DLCT-based SPR prediction in this study showed similar accuracy compared to other DECT systems for SPR prediction (12–14, 40). The SPR values of certain non-tissue implant materials used in this work have been previously investigated using dual-source CT (14). The DLCT functionality of the Philips Spectral CT 7500 yielded a similar SPR prediction accuracy as the dual-source CT in this previous study.

### 4.3 Clinical Relevance

As CT technology continues to improve, scanners with DECT capabilities are becoming increasingly available—they are already used for diagnostic purposes at many healthcare facilities. The application of DECT to particle therapy treatment planning could potentially improve patient outcomes. For example, inaccuracies in SPR prediction for pediatric proton therapy planning arising from SECT calibration curves based on adult male tissues may be avoided with DECT (41). Furthermore, SECT-based SPR prediction has been shown to introduce large inter-center variations in SPR, reaching up to 9% between different European institutions (42). Thus, DECT-based SPR prediction might offer more consistent SPR predictions between treatment centers or allow new particle therapy centers to begin treatment with greater confidence in SPR prediction. Moreover, recent work has demonstrated the benefits of even small reductions in range uncertainty to normal tissue complication probability (43), supporting that even small improvements in SPR prediction may be clinically beneficial.

### 4.4 Study Limitations and Future Work

This study demonstrated the feasibility of direct, patient-specific SPR prediction using existing clinical equipment and frameworks. However, in order to use DLCT for SPR prediction beyond a defined research environment, it will be necessary to devise and implement a complete workflow of certified medical products which does not currently exist. To start, SPR DICOM files could be provided as an on-demand spectral result directly from the Philips Spectral CT 7500 scanner instead of needing additional calculation steps using ED and EAN data.

The strengths and limitations of the different DECT or spectral CT acquisition techniques currently available have been described in previous works (7, 44–46) and are summarized in **Supplementary Table 1**, with a focus on applications to particle therapy treatment planning. Additionally, **Supplementary Table 2** lists selected publications on the different DECT or spectral CT acquisition techniques to provide an overview of the current state of research. The optimal DECT acquisition technique and hardware choice depends on the purpose of the application (e.g., body site, presence of motion) and the relative effect of various parameters (e.g., spectral separation, impact of scattering, tube current modulation) (7), which makes it difficult to give a general recommendation. Imaging with a dual-layer detector enables perfectly temporally and spatially aligned data sets. Moreover, DLCT imaging allows for tube current modulation, a full scan field-of-view coverage, and requires no special mode for DECT acquisition. The dual-layer detector design also enables acquisition of dual-energy data at exactly the same phase of contrast enhancement. Furthermore, the DLCT technique facilitates projection-based material decomposition, allowing for better noise reduction and therefore potentially better material decomposition as compared to image-based methods (47). Nevertheless, spectral separation of DLCT systems is lower than that of source-based DECT systems (44), and spectral signal-to-noise ratio is comparable to that of other commercial DECT systems (48). In addition, DLCT systems carry the risk of cross-scatter occurring between detector layers (45).

In the future, particle CT might have the potential to further improve SPR prediction accuracies and serve as a ground-truth when comparing DECT-based SPR predictions (49). Thus far, precise SPR measurements using proton CT or helium CT are challenging to achieve, and provide a slightly lower SPR prediction accuracy compared to DECT (50).

While the tissue-equivalent materials used in this study are considered valid surrogates for biological tissues, they cannot fully represent the heterogeneity and variable composition of real tissues. Before DLCT-based SPR prediction can be implemented in clinical practice, more studies on biological tissue samples and *in vivo* systems need to be performed. In addition, measurements in this study were only performed with a male pelvic phantom, introducing a gender data gap. It would be desirable to perform similar measurements with a female pelvic phantom, but at the present time such a phantom does not exist. Furthermore, 4D treatment planning is important for radiotherapy treatments which require motion mitigation and/or consideration during treatment, such as the thorax and the abdominal region. Future work could implement 4D DLCT-based SPR prediction and treatment planning. The large coverage of the Philips Spectral CT 7500 compared to the Philips IQon Spectral CT (80 mm versus 40 mm) (**Supplementary Table 3**) means that a larger portion of the patient anatomy is covered per gantry rotation of the CT system, leading to potential reduction in motion artifacts. Combining this feature with DLCT-based SPR prediction may enhance 4D CT planning in particle therapy for moving targets.

Finally, other beneficial characteristics of DECT should be investigated for all technical implementations available, including DLCT, to understand the full advantages of the technology. Beyond the computational aspects of DECT-based treatment planning discussed in this work, DECT imaging is expected to provide various opportunities to improve the accuracy of multiple parts of the radiotherapy chain. DECT has been suggested to improve image quality and reduce metal artifacts (51), to improve tumor staging, delineation, and characterization (52, 53), and to contribute to improved normal tissue characterization and personalized treatment through physiological quantification (46). Furthermore, DECT also shows potential for improved dose calculations for treatment modalities other than particle therapy, such as brachytherapy and conventional photon-based teletherapy (51). Finally, as briefly explored in this study and proposed by Albrecht et al. (54), DECT has the potential to reduce total imaging dose. Future work might investigate these varied applications of DECT to radiotherapy.

## Data Availability Statement

The raw data supporting the conclusions of this article will be made available by the authors, without undue reservation.

## Author Contributions

Conceptualization, FL, AK, AM, and WS. Methodology, FL, AK, AM, and WS. Data acquisition and analysis, FL, AK, and BA. Interpretation, FL, AK, SM, TT, AM, and WS. Writing—original draft preparation, FL and AK. Writing—review and editing, FL, AK, SM, TT, BA, JD, AM, and WS. Supervision, JD, AM, and WS. All authors contributed to the article and approved the submitted version.

## Funding

For the publication fee we acknowledge financial support by Deutsche Forschungsgemeinschaft within the funding programme "Open Access Publikationskosten" as well as by Heidelberg University.

## Conflict of Interest

WS is a member of the CT Advisory Board of Philips Medical Systems.

The remaining authors declare that the research was conducted in the absence of any commercial or financial relationships that could be construed as a potential conflict of interest.

## Publisher’s Note

All claims expressed in this article are solely those of the authors and do not necessarily represent those of their affiliated organizations, or those of the publisher, the editors and the reviewers. Any product that may be evaluated in this article, or claim that may be made by its manufacturer, is not guaranteed or endorsed by the publisher.
