# Supplementary material for: Potential of a Second-Generation Dual-Layer Spectral CT for Dose Calculation in Particle Therapy Treatment Planning
Source: Front Oncol. 2022 Apr 20;12:853495. doi: 10.3389/fonc.2022.853495 (PMC9069208; doi:10.3389/fonc.2022.853495)
Supplement: Supplementary file 1 [file DataSheet_1.pdf]

Supplementary Material

1 Supplementary Figures and Tables

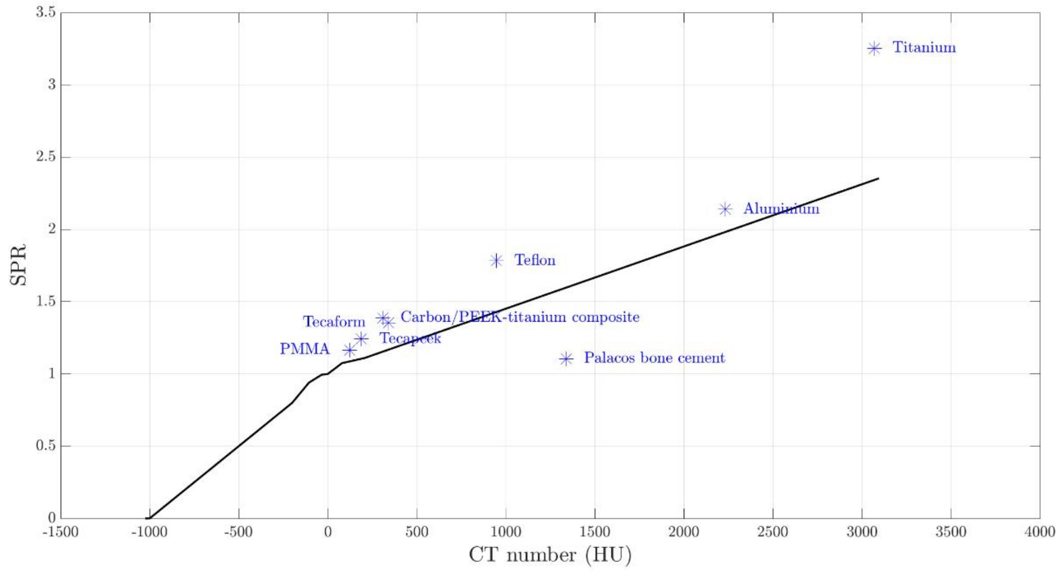

Supplementary Figure 1. Single-energy CT (SECT)-based CT-number-to-stopping-power-ratio (SPR) calibration curve, or Hounsfield look-up table (HLUT), together with the eight non-tissue implant materials used in this study.

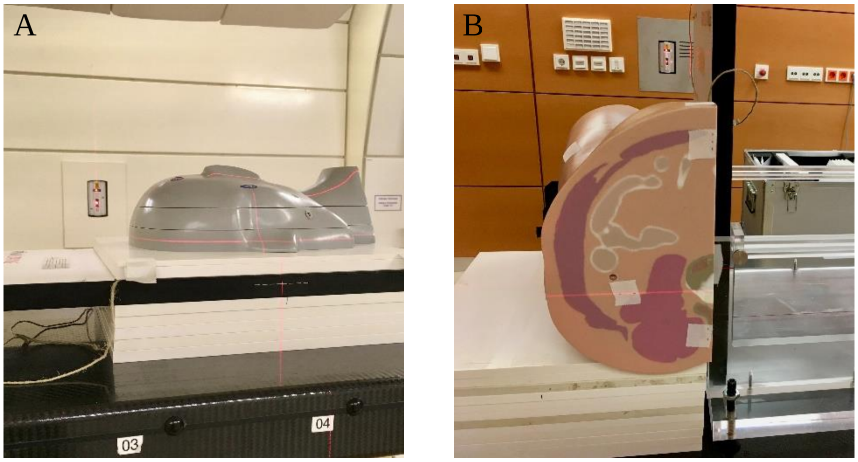

Supplementary Figure 2. Dosimetric measurements with anthropomorphic head and pelvic phantoms. For the head phantom (A), irradiation was performed using the gantry at an angle of 0° with the half-head phantom placed on top of the OCTAVIUS® detector. For the pelvic phantom (B), irradiation was performed using the horizontal beam line with the half-pelvic phantom placed in front of the OCTAVIUS® detector.

Technical features	Dual-source CT	Dual-spiral	Fast tube voltage switching	Twin-beam	Dual-layer detector	Photon-counting CT
**Spectral mode**	spectral mode preselection, prospective results generation	spectral mode preselection, prospective results generation	spectral mode preselection, prospective results generation	spectral mode preselection, prospective results generation	no special mode, pro- and retrospective results generation	no special mode, pro- and retrospective results generation
**Acquisition mode**	dedicated dual-energy	dedicated dual-energy	dedicated dual-energy	dedicated dual-energy	implicit dual-energy	implicit multi-energy
**Temporal coherence**	high (quarter rotation angular offset)	low (large offset)	high (nearly perfect alignment)	medium (half collimation offset)	perfect alignment	perfect alignment
**Spatio-temporal resolution**	full capabilities	full capabilities	limited capabilities (impaired spatial resolution)	full capabilities	full capabilities	full capabilities (superior spatial resolution)
**Availability of dose modulation**	yes (individual tube current modulation)	yes (individual tube current modulation)	no (no tube current modulation)	yes (tube current modulation)	yes (tube current modulation)	yes (tube current modulation)
**Spectral separation**	very high (with additional tin filtration)	very high	medium ("smearing of spectra" due to finite kV-switching times)	low	medium	high
**Cross-scatter occurrence**	between source–detector systems	no	no	between detector rows (halves) separated by the system's central vertical plane	between detector layers	no
**Field-of-view**	limited (up to 350 mm)	full	full	full	full	full

**Time-resolved respiratory imaging**	feasible with phase matching in post-processing	feasible with phase matching in post-processing	feasible with phase matching in post-processing	feasible with phase matching in post-processing	no technical limitations	no technical limitations
**Contrast-enhanced imaging**	multi-phase (arterial and venous)	limited to late or delayed phase	multi-phase (arterial and venous)	limited to late or delayed phase	multi-phase (arterial and venous)	multi-phase (arterial and venous)
**Spectral decompo-sition**	image-based material decomposition	image-based material decomposition	projection-based material decomposition	image-based material decomposition	projection-based material decomposition	projection-based material decomposition

Supplementary Table 1. Comparison of different dual-energy or spectral CT acquisition techniques with a focus on particle therapy treatment planning. Note that despite having the potential to provide spectral data, CT systems with energy-resolving, photon-counting detectors are just starting to become clinically available but do not yet see widespread use.

	Dual-source CT	Dual-spiral	Fast tube voltage switching	Twin-beam	Dual-layer detector	Photon-counting CT
**Theoretical studies**	• [4] Bär et al (2017)					• [27] Taasti et al (2018)
**Phantom studies (phantoms with simplified geometries or anthropo-morphic phantoms)**	• [2] Almeida et al (2018)<br>• [4] Bär et al (2017)<br>• [5] Bär et al (2018)<br>• [7] Bourque et al (2014)<br>• [10] Hansen et al (2015)<br>• [11] Hudobivnik et al (2016)<br>• [12] Hünemohr et al (2014)<br>• [15] Li et al (2017)<br>• [17] Michalak et al (2017)<br>• [23] Saito et al (2017)<br>• [29] Wohlfahrt et al (2018)	• [1] Almeida et al (2017)<br>• [2] Almeida et al (2018)<br>• [8] Chacko et al (2021)<br>• [19] Mossahebi et al (2020)<br>• [24] Shen et al (2018)<br>• [33] Zhang et al (2019)<br>• [34] Zhu & Penfold (2016)	• [21] Ohira et al (2022)	• [2] Almeida et al (2018)	• [9] Faller et al (2020)<br>• [13] Landry et al (2019)<br>• [20] Ohira et al (2018)	• [14] Lee et al (2021)
**Biological tissue sample studies (homo-geneous or hetero-geneous tissue samples)**	• [5] Bär et al (2018)<br>• [18] Möhler et al (2018)<br>• [25] Taasti et al (2017)	• [25] Taasti et al (2017)<br>• [32] Xie et al (2018)		• [25] Taasti et al (2017)		• [27] Taasti et al (2018)

**Patient studies**	• [6] Bär et al (2021)<br>• [11] Hudobivnik et al (2016)<br>• [26] Taasti et al (2018)	• [2] Almeida et al (2018)<br>• [22] Peters et al (2021)<br>• [26] Taasti et al (2018)<br>• [28] Wohlfahrt et al (2017)<br>• [30] Wohlfahrt et al (2018)<br>• [31] Wohlfahrt et al (2019)			• [3] Ates et al (2021)<br>• [16] Longarino et al (2022)	

Supplementary Table 2. Selected publications of different dual-energy or spectral CT acquisition techniques currently available with a focus on particle therapy treatment planning. Note: The table is in alphabetical order and might not be exhaustive.

[1] Almeida et al (2017): Siemens SOMATOM Definition Open AS
[2] Almeida et al (2018): Siemens SOMATOM Force, Siemens SOMATOM Confidence RT Pro, Siemens SOMATOM Definition Edge
[3] Ates et al (2021): Philips IQon Spectral CT
[4] Bär et al (2017): Siemens SOMATOM Definition Flash
[5] Bär et al (2018): Siemens SOMATOM Definition Flash
[6] Bär et al (2021): Siemens SOMATOM Definition Flash
[7] Bourque et al (2014): Siemens SOMATOM Definition Flash
[8] Chacko et al (2021): Siemens SOMATOM Confidence RT Pro
[9] Faller et al (2020): Philips IQon Spectral CT
[10] Hansen et al (2015): Siemens SOMATOM Definition Flash
[11] Hudobivnik et al (2016): Siemens SOMATOM Force
[12] Hünemohr et al (2014): Siemens SOMATOM Definition Flash
[13] Landry et al (2019): Philips IQon Spectral CT
[14] Lee et al (2021): Energy-differentiation-type 64-channel cadmium telluride (CdTe) radiation line sensor module (C10413, Hamamatsu, Japan)
[15] Li et al (2015): Siemens SOMATOM Force
[16] Longarino et al (2022): Philips IQon Spectral CT
[17] Michalak et al (2017): Siemens SOMATOM Force
[18] Möhler et al (2018): Siemens SOMATOM Definition Flash
[19] Mossahebi et al (2020): Siemens SOMATOM Definition Edge
[20] Ohira et al (2018): Philips IQon Spectral CT
[21] Ohira et al (2022): GE Healthcare Revolution HD
[22] Peters et al (2021): Siemens SOMATOM Definition AS
[23] Saito et al (2017): Siemens SOMATOM Definition Flash
[24] Shen et al (2018): GE Healthcare LightSpeed QX/i
[25] Taasti et al (2017): Siemens SOMATOM Definition AS, Siemens SOMATOM Definition Flash, Siemens SOMATOM Force, Siemens SOMATOM Definition Edge
[26] Taasti et al (2018): Siemens SOMATOM Definition Flash, Philips Brilliance Big Bore
[27] Taasti et al (2018): Siemens research SOMATOM CounT
[28] Wohlfahrt et al (2017): Siemens SOMATOM Definition AS
[29] Wohlfahrt et al (2018): Siemens SOMATOM Definition AS
[30] Wohlfahrt et al (2018): Siemens SOMATOM Definition AS
[31] Wohlfahrt et al (2019): Siemens SOMATOM Definition AS
[32] Xie et al (2018): Siemens SOMATOM Sensation Open
[33] Zhang et al (2019): Philips Brilliance Big Bore
[34] Zhu & Penfold (2016): Philips Brilliance Big Bore

Feature	Spectral CT 7500	IQon Spectral CT
Generator power	120 kW	120 kW
Maximum detector collimation	128 × 0.625 mm	64 × 0.625 mm
Coverage (per rotation)	80 mm	40 mm
Minimum gantry rotation time	0.27 s	0.27 s
Maximum scannable range (axial)	2000 mm	2100 mm
Bore size	800 mm	700 mm
Conventional reconstruction time	iDose^4: 93% of reference protocols under 1 minute	iDose^4: majority of reference protocols under 1 minute
Spectral reconstruction time	1–2 minutes for the majority of cases	3–5 minutes for the majority of cases
Spectral temporal resolution	Simultaneous in the same time and space	Simultaneous in the same time and space
Spectral kV_p stations	100, 120, 140	120, 140

Supplementary Table 3. Comparison of the Philips Spectral CT 7500 [35] and IQon Spectral CT [36] scanners.

Protocol	Tube voltage (kV_p)	Tube current-time product (mAs)	Colli-mation (mm)	Rotation time (s)	Pitch	$CTDI_{vol}$ (mGy)	Slice thickness and spacing (mm)	Recon-struction filter
Head	120	300	64 × 0.625	0.5	0.8	47.2	1.5	UB
Body	120	300	128 × 0.625	0.5	0.8	23.2	2.0	B

Supplementary Table 4. Image acquisition settings and reconstruction parameters for head and body protocols.

**Phantom**	**LC**				**SC**			
Protocol	Head	Body			Head	Body		
iDose[4] level	0	0	3	6	0	0	3	6
Mean overall relative residual	0.728	0.725	0.723	0.724	0.761	0.613	0.579	0.605
RMSE	0.0086	0.0084	0.0085	0.0084	0.0089	0.0056	0.0056	0.0057
r	0.9998	0.9998	0.9998	0.9998	0.9998	0.9998	0.9998	0.9998
α	1.016	1.011	1.011	1.001	1.015	1.005	1.005	1.005
δ	–0.012	–0.006	–0.006	–0.006	–0.010	–0.002	–0.001	–0.002

Supplementary Table 5. Accuracy of dual-layer spectral CT (DLCT)-based stopping power ratio (SPR) predictions across head and body protocols and iDose[4] levels 0, 3, and 6 for the LC (“long cylinder”) phantom and SC (“short cylinder”) phantom.

**Phantom**	**LC**				**SC**			
Protocol	Head	Body			Head	Body		
iDose[4] level	0	0	3	6	0	0	3	6
Mean overall relative residual	1.514	1.538	1.540	1.537	1.515	1.514	1.523	1.516
RMSE	0.0246	0.0255	0.0256	0.0256	0.0240	0.0243	0.0244	0.0243
r	0.9958	0.9956	0.9956	0.9956	0.9959	0.9958	0.9958	0.9958
α	1.009	1.012	1.012	1.012	1.005	1.007	1.008	1.008
δ	–0.009	–0.009	–0.009	–0.009	–0.007	–0.008	–0.008	–0.008

Supplementary Table 6. Accuracy of single-energy CT (SECT)-based stopping power ratio (SPR) predictions across head and body protocols and iDose[4] levels 0, 3, and 6 for the LC (“long cylinder”) phantom and SC (“short cylinder”) phantom.

2 References

[1] Almeida IP, Landry G, Dedes G, Patel R, Pankuch M, Coutrakon G et al. Evaluating clinical stopping power estimation from a radiotherapy dual energy CT scanner. Acta Phys Pol B (2017) 48(10):1619-1623. doi:10.5506/APhysPolB.48.1619

[2] Almeida IP, Schyns LEJR, Vaniqui A, van der Heyden B, Dedes G, Resch AF et al. Monte Carlo proton dose calculations using a radiotherapy specific dual-energy CT scanner for tissue segmentation and range assessment. Phys Med Biol (2018) 63(11):115008. doi: 10.1088/1361-6560/aabb60

[3] Ates O, Hua CH, Zhao L, Shapira N, Yagil Y, Merchant TE et al. Feasibility of using post-contrast dual-energy CT for pediatric radiation treatment planning and dose calculation. Br J Radiol (2021) 94(1118):20200170. doi: 10.1259/bjr.20200170

[4] Bär E, Lalonde A, Royle G, Lu HM, Bouchard H. The potential of dual-energy CT to reduce proton beam range uncertainties. Med Phys (2017) 44(6):2332-2344. doi: 10.1002/mp.12215

[5] Bär E, Lalonde A, Zhang R, Jee KW, Yang K, Sharp G et al. Experimental validation of two dual-energy CT methods for proton therapy using heterogeneous tissue samples. Med Phys (2018) 45(1):48-59. doi: 10.1002/mp.12666

[6] Bär E, Collins-Fekete CA, Rompokos V, Zhang Y, Gaze MN, Warry A et al. Assessment of the impact of CT calibration procedures for proton therapy planning on pediatric treatments. Med Phys (2021) 48(9):5202-5218. doi: 10.1002/mp.15062

[7] Bourque AE, Carrier JF, Bouchard H. A stoichiometric calibration method for dual energy computed tomography. Phys Med Biol (2014) 59(8):2059-2088. doi: 10.1088/0031-9155/59/8/2059

[8] Chacko MS, Grewal HS, Wu D, Sonnad JR. Accuracy of proton stopping power estimation of silicone breast implants with single and dual-energy CT calibration techniques. J Appl Clin Med Phys (2021) 22(9):159-170. doi: 10.1002/acm2.13358

[9] Faller FK, Mein S, Ackermann B, Debus J, Stiller W, Mairani A. Pre-clinical evaluation of dual-layer spectral computed tomography-based stopping power prediction for particle therapy planning at the Heidelberg Ion Beam Therapy Center. Phys Med Biol (2020) 65(9):095007. doi: 10.1088/1361-6560/ab735e

[10] Hansen DC, Seco J, Sørensen TS, Petersen JB, Wildberger JE, Verhaegen F et al. A simulation study on proton computed tomography (CT) stopping power accuracy using dual energy CT scans as benchmark. Acta Oncol (2015) 54(9):1638-1642. doi: 10.3109/0284186X.2015.1061212

[11] Hudobivnik N, Schwarz F, Johnson T, Agolli L, Dedes G, Tessonnier T et al. Comparison of proton therapy treatment planning for head tumors with a pencil beam algorithm on dual and single energy CT images. Med Phys (2016) 43(1):495-504. doi: 10.1118/1.4939106

[12] Hünemohr N, Krauss B, Tremmel C, Ackermann B, Jäkel O, Greilich S. Experimental verification of ion stopping power prediction from dual energy CT data in tissue surrogates. Phys Med Biol (2014) 59(1):83-96. doi: 10.1088/0031-9155/59/1/83

[13] Landry G, Dörringer F, Si-Mohamed S, Douek P, Abascal JFPJ, Peyrin F et al. Technical Note: Relative proton stopping power estimation from virtual monoenergetic images reconstructed from dual-layer computed tomography. Med Phys (2019) 46(4):1821-1828. doi: 10.1002/mp.13404

[14] Lee SH, Sunaguchi N, Nagao A, Hirano Y, Sakurai H, Kano Y et al. Calculation of Stopping-Power Ratio from Multiple CT Numbers Using Photon-Counting CT System: Two- and Three-Parameter-Fitting Method. Sensors (2021) 21(4):1215. doi: 10.3390/s21041215

[15] Li B, Lee HC, Duan X, Shen C, Zhou L, Jia X et al. Comprehensive analysis of proton range uncertainties related to stopping-power-ratio estimation using dual-energy CT imaging. Phys Med Biol (2017) 62(17):7056-7074. doi: 10.1088/1361-6560/aa7dc9

[16] Longarino FK, Tessonnier T, Mein S, Harrabi SB, Debus J, Stiller W et al. Dual-layer spectral CT for proton, helium, and carbon ion beam therapy planning of brain tumors. J Appl Clin Med Phys (2022) 23(1):e13465. doi: 10.1002/acm2.13465

[17] Michalak G, Taasti V, Krauss B, Deisher A, Halaweish A, McCollough C. A comparison of relative proton stopping power measurements across patient size using dual- and single-energy CT. Acta Oncol (2017) 56(11):1465-1471. doi: 10.1080/0284186X.2017.1372625

[18] Möhler C, Russ T, Wohlfahrt P, Elter A, Runz A, Richter C et al. Experimental verification of stopping-power prediction from single- and dual-energy computed tomography in biological tissues. Phys Med Biol (2018) 63(2):025001. doi: 10.1088/1361-6560/aaa1c9

[19] Mossahebi S, Sabouri P, Chen H, Mundis M, O'Neil M, Maggi P et al. Initial Validation of Proton Dose Calculations on SPR Images from DECT in Treatment Planning System. Int J Part Ther (2020) 7(2):51-61. doi: 10.14338/IJPT-XX-000XX.1

[20] Ohira S, Washio H, Yagi M, Karino T, Nakamura K, Ueda Y et al. Estimation of electron density, effective atomic number and stopping power ratio using dual-layer computed tomography for radiotherapy treatment planning. Physica Medica (2018) 56:34-40. doi: 10.1016/j.ejmp.2018.11.008

[21] Ohira S, Imai Y, Koike Y, Ono S, Ueda Y, Miyazaki M et al. Evaluation of Stopping Power Ratio Calculation Using Dual-energy Computed Tomography With Fast Kilovoltage Switching for Treatment Planning of Particle Therapy. In Vivo (2022) 36(1):103-110. doi: 10.21873/invivo.12681

[22] Peters N, Wohlfahrt P, Hofmann C, Möhler C, Menkel S, Tschiche M et al. Reduction of clinical safety margins in proton therapy enabled by the clinical implementation of dual-energy CT for direct stopping-power prediction. Radiother Oncol (2021) 166:71-78. doi: 10.1016/j.radonc.2021.11.002

[23] Saito M, Sagara S. Simplified derivation of stopping power ratio in the human body from dual-energy CT data. Med Phys (2017) 44(8):4179-4187. doi: 10.1002/mp.12386

[24] Shen C, Li B, Chen L, Yang M, Lou Y, Jia X. Material elemental decomposition in dual and multi-energy CT via a sparsity-dictionary approach for proton stopping power ratio calculation. Med Phys (2018) 45(4):1491-1503. doi: 10.1002/mp.12796

[25] Taasti VT, Michalak GJ, Hansen DC, Deisher AJ, Kruse JJ, Krauss B et al. Validation of proton stopping power ratio estimation based on dual energy CT using fresh tissue samples. Phys Med Biol (2017) 63(1):015012. doi: 10.1088/1361-6560/aa952f

[26] Taasti VT, Muren LP, Jensen K, Petersen JBB, Thygesen J, Tietze A et al. Comparison of single and dual energy CT for stopping power determination in proton therapy of head and neck cancer. Phys Imaging Radiat Oncol (2018) 6:14-19. doi: 10.1016/j.phro.2018.04.002

[27] Taasti VT, Hansen DC, Michalak GJ, Deisher AJ, Kruse JJ, Muren LP et al. Theoretical and experimental analysis of photon counting detector CT for proton stopping power prediction. Med Phys (2018) 45(11):5186-5196. doi: 10.1002/mp.13173

[28] Wohlfahrt P, Möhler C, Stützer K, Greilich S, Richter C. Dual-energy CT based proton range prediction in head and pelvic tumor patients. Radiother Oncol (2017) 125(3):526-533. doi: 10.1016/j.radonc.2017.09.042

[29] Wohlfahrt P, Möhler C, Richter C, Greilich S. Evaluation of Stopping-Power Prediction by Dual- and Single-Energy Computed Tomography in an Anthropomorphic Ground-Truth Phantom. Int J Radiat Oncol Biol Phys (2018) 100(1):244-253. doi: 10.1016/j.ijrobp.2017.09.025

[30] Wohlfahrt P, Troost EGC, Hofmann C, Richter C, Jakobi A. Clinical Feasibility of Single-Source Dual-spiral 4D Dual-Energy CT for Proton Treatment Planning Within the Thoracic Region. Int J Radiat Oncol Biol Phys (2018) 102(4):830-840. doi: 10.1016/j.ijrobp.2018.06.044

[31] Wohlfahrt P, Möhler C, Troost EGC, Greilich S, Richter C. Dual-Energy Computed Tomography to Assess Intra- and Inter-Patient Tissue Variability for Proton Treatment Planning of Patients With Brain Tumor. Int J Radiat Oncol Biol Phys (2019) 105(3):504-513. doi: 10.1016/j.ijrobp.2019.06.2529

[32] Xie Y, Ainsley C, Yin L, Zou W, McDonough J, Solberg TD et al. Ex vivo validation of a stoichiometric dual energy CT proton stopping power ratio calibration. Phys Med Biol (2018) 63(5):055016. doi: 10.1088/1361-6560/aaae91

[33] Zhang S, Han D, Williamson JF, Zhao T, Politte DG, Whiting BR et al. Experimental implementation of a joint statistical image reconstruction method for proton stopping power mapping from dual-energy CT data. Med Phys (2019) 46(1):273-285. doi: 10.1002/mp.13287

[34] Zhu J, Penfold SN. Dosimetric comparison of stopping power calibration with dual-energy CT and single-energy CT in proton therapy treatment planning. Med Phys (2016) 43(6):2845-2854. doi: 10.1118/1.4948683

[35] Philips. Spectral CT 7500 (2021). URL: https://www.usa.philips.com/healthcare/product/728333/spectral-ct-7500-philips-all-new-spectral-detector-ct-750 [accessed November 22, 2021]

[36] Philips. IQon Spectral CT (2021). URL: https://www.usa.philips.com/healthcare/product/HCNOCTN284/iqon-spectral-ct-certainty-lives-in-layers [accessed November 22, 2021]
